# Analysis of Female Enrollment and Participant Sex by Burden of Disease in US Clinical Trials Between 2000 and 2020

**DOI:** 10.1001/jamanetworkopen.2021.13749

**Published:** 2021-06-18

**Authors:** Jecca R. Steinberg, Brandon E. Turner, Brannon T. Weeks, Christopher J. Magnani, Bonnie O. Wong, Fatima Rodriguez, Lynn M. Yee, Mark R. Cullen

**Affiliations:** 1 Department of Obstetrics and Gynecology, Northwestern Feinberg School of Medicine, Chicago, Illinois; 2 Harvard Radiation Oncology Program, Massachusetts General Hospital and the Joint Center for Radiation Therapy, Boston; 3 Department of Obstetrics and Gynecology, Stanford University School of Medicine, Stanford, California; 4 Division of Cardiovascular Medicine and the Cardiovascular Institute, Stanford University, Stanford, California; 5 Center for Population Health Sciences, Stanford University School of Medicine, Palo Alto, California

## Abstract

**Importance:**

Although female representation has increased in clinical trials, little is known about how clinical trial representation compares with burden of disease or is associated with clinical trial features, including disease category.

**Objective:**

To describe the rate of sex reporting (ie, the presence of clinical trial data according to sex), compare the female burden of disease with the female proportion of clinical trial enrollees, and investigate the associations of disease category and clinical trial features with the female proportion of clinical trial enrollees.

**Design, Setting, and Participants:**

This cross-sectional study included descriptive analyses and logistic and generalized linear regression analyses with a logit link. Data were downloaded from the Aggregate Analysis of ClinicalTrials.gov database for all studies registered between March 1, 2000, and March 9, 2020. Enrollment was compared with data from the 2016 Global Burden of Disease database. Of 328 452 clinical trials, 70 095 were excluded because they had noninterventional designs, 167 936 because they had recruitment sites outside the US, 69 084 because they had no reported results, 1003 because they received primary funding from the US military, and 314 because they had unclear sex categories. A total of 20 020 interventional studies enrolling approximately 5.11 million participants met inclusion criteria and were divided into those with and without data on participant sex.

**Exposures:**

The primary exposure variable was clinical trial disease category. Secondary exposure variables included funding, study design, and study phase.

**Main Outcomes and Measures:**

Sex reporting and female proportion of participants in clinical trials.

**Results:**

Among 20 020 clinical trials from 2000 to 2020, 19 866 studies (99.2%) reported sex, and 154 studies (0.8%) did not. Clinical trials in the fields of oncology (46% of disability-adjusted life-years [DALYs]; 43% of participants), neurology (56% of DALYs; 53% of participants), immunology (49% of DALYs; 46% of participants), and nephrology (45% of DALYs; 42% of participants) had the lowest female representation relative to corresponding DALYs. Male participants were underrepresented in 8 disease categories, with the greatest disparity in clinical trials of musculoskeletal disease and trauma (11.3% difference between representation and proportion of DALYs). Clinical trials of preventive interventions were associated with greater female enrollment (adjusted relative difference, 8.48%; 95% CI, 3.77%-13.00%). Clinical trials in cardiology (adjusted relative difference, −18.68%; 95% CI, −22.87% to −14.47%) and pediatrics (adjusted relative difference, −20.47%; 95% CI, −25.77% to −15.16%) had the greatest negative association with female enrollment.

**Conclusions and Relevance:**

In this study, sex differences in clinical trials varied by clinical trial disease category, with male and female participants underrepresented in different medical fields. Although sex equity has progressed, these findings suggest that sex bias in clinical trials persists within medical fields, with negative consequences for the health of all individuals.

## Key Points

**Question:**

How is disease burden associated with female representation in US-based clinical trials, and how are disease category and other clinical trial features associated with female representation in clinical trials?

**Findings:**

In this cross-sectional study of 20 020 clinical trials enrolling more than 5 million participants between 2000 and 2020, clinical trials in the fields of oncology, neurology, immunology, and nephrology had the lowest female participant representation relative to corresponding disability-adjusted life-years. Clinical trials in cardiology and pediatrics had the greatest negative associations with female enrollment, and clinical trials of preventive interventions had a positive association.

**Meaning:**

This study’s findings suggest that sex bias persists within clinical trials, with male and female participants underrepresented in different areas of research.

## Teaser

This cross-sectional study uses data from clinical trials registered in ClinicalTrials.gov between 2000 and 2020 to assess the presence of clinical trial data according to sex, female burden of disease relative to female proportion of enrollees, and associations of disease category and study features with female proportion of enrollees.

## Introduction

Medical research has historically focused on male health.^1,2,3,4,5^ Female individuals were often excluded from clinical trials, supposedly to ensure homogeneity of treatment effect and reduce potential maternal-fetal liability.^6^ Sex bias persisted, even after research reported sex differences in diagnostic test results, disease progression, treatment response, drug metabolism, and surgical outcomes.^7^ Studies have associated this lack of female inclusion with suboptimal health care and adverse medical outcomes.^4,8^ In response to the recognition of sex bias in clinical trials and corresponding health disparities, national and international organizations have mobilized legislative efforts to increase female inclusion.^9^ Research documented the ensuing increase in female representation in clinical trials and dismantled inaccurate notions that female participants complicated results by introducing confounding social and biological variables.^9^ By 2013, female enrollees comprised more than one-half of all participants in National Institutes of Health research.^9^

Most studies on sex bias in research have focused on equality and sex matching, pairing female representation with male representation.^5,10^ Sex matching and power for sex-specific analysis permit study stratification and the detection of effect differences between male and female participants. However, these parameters may not adequately capture heterogeneity or adverse events in subpopulations because they rarely account for the varied distributions and manifestations of disease within sexes.^11^ Greater emphasis on parity and representation, not simply statistical power, has emerged with the increasing interest in pragmatic clinical trials and clinical trials that evaluate effectiveness rather than efficacy.^11,12^ Despite the inherent association between representation and burden of disease, little is known about how the sex of clinical trial participants compares with disease prevalence.^13^

We aimed to address this gap by characterizing sex reporting (ie, the presence of clinical trial data on the number of male and female participants) in US-based clinical trials that were registered in the ClinicalTrials.gov database between 2000 and 2020 and by comparing sex representation in clinical trials with the burden of disease by sex. We also investigated the associations of clinical trial funding, clinical trial phase, and other clinical trial features with female representation across medical disciplines.

## Methods

### Data Sources

We downloaded all clinical trials registered between March 1, 2000, and March 9, 2020, to ClinicalTrials.gov using the Aggregate Analysis of ClinicalTrials.gov (AACT) database.^14^ Observational studies were excluded. Although the ClinicalTrials.gov registry captures most US-based clinical trials, many international clinical trials are registered in other international or country-specific databases. Thus, we limited our study to US-based clinical trials to ensure accurate comparisons between the clinical trial sample and the represented population. The analysis included all studies that (1) were registered in ClinicalTrials.gov; (2) were conducted between March 1, 2000, and March 9, 2020; (3) reported sex results; and (4) were interventional in design. We referenced 2016 Global Burden of Disease (GBD) data to assess the US burden of disease for each sex.^15^ This study followed the Strengthening the Reporting of Observational Studies in Epidemiology (STROBE) reporting guideline for cross-sectional studies.^16^ The study was reviewed and approved by the institutional review board of Stanford University and granted exemption from informed consent because the study did not involve human participants and included only publicly available data sets.

### Exposure Variables

Primary exposure variables included clinical trial disease category and clinical trial funding. Secondary analyses investigated clinical trial phase and other clinical trial features.

We defined burden of disease as disability-adjusted life-years (DALYs; ie, years of healthy life lost) based on GBD data.^15^ We selected DALYs rather than prevalence because DALYs capture the varied sequelae and burden of disease by sex. We combined medical subject heading (MeSH) terms and GBD information by manually assigning diseases in the GBD database (eMethods 1 in the Supplement) to 1 or more of the 17 most prevalent MeSH disease categories in the US population: (1) musculoskeletal and trauma, (2) psychiatry, (3) cardiology, (4) neurology (including ocular disease), (5) oncology, (6) pulmonology, (7) pediatrics, (8) endocrinology, (9) gastroenterology, (10) nutritional and metabolic, (11) infectious disease, (12) immunology, (13) otorhinolaryngology (including stomatognathic disease), (14) dermatology, (15) nephrology and genitourinary (subdivided into male-specific, female-specific, and sex-nonspecific), (16) hematology (hemic and lymphatic disease), and (17) congenital. Categories were not exclusive because given diseases may have implications for multiple organ systems, and they were modeled as binary variables in the analysis.^17^

We classified funding in accordance with previous studies on the AACT database,^17,18^ using both the lead sponsor and collaborating agencies. Industry-funded clinical trials included all studies with an industry source as a sponsor or collaborator. Remaining clinical trials with a US government funder were labeled *US government*. Given that the US military is predominantly male, we excluded clinical trials with military funding. Remaining clinical trials (listed as *other* in the AACT database) were labeled *academic*, as 90% of the funders in the *other* category have been reported in previous studies to be academic institutions.^18,19^

Clinical trial features included (1) primary purpose, (2) intervention, (3) phase, (4) number of arms, (5) estimated enrollment, (6) registration year, (7) blinding, (8) randomization, (9) placebo control, (10) control with active comparator, (9) data safety monitoring committee oversight, (11) number of sites, and (12) study status.

### Outcomes

The primary outcomes were sex reporting and the proportion of female participants in clinical trials. Sex was defined according to the biological definitions of male and female. Although there are inherent associations between sex and gender (a cultural and psychosocial term that captures the complex manifestations associated with sex in a societal context^20^), we exclusively focused on sex as reported in the AACT and GBD databases. In the AACT database, sex and gender can be entered as *sex, male, female* and/or *gender, customized*. More than 45 terms characterize gender in the database. Using both manual and programmatic searches, we identified and extracted clinical trials that reported sex or customized gender enrollment data. Two authors (B.E.T. and B.O.W.) manually reviewed all gender-customized labels and sorted them into 2 categories, male or female, in accordance with the enrollees’ biological sex. Clinical trials with unintelligible sex reporting that could not be characterized as male or female (ie, incorrectly completed fields) were excluded. Although the ClinicalTrials.gov registry only developed a reporting repository for enrollee demographic data in 2007,^21^ we included clinical trials registered before 2007 if they had reported results in the AACT database.

### Statistical Analysis

We aimed to (1) describe the rate of sex reporting, (2) compare the female burden of disease with the proportion of female clinical trial enrollees, (3) characterize the proportion of female enrollees by clinical trial features, (4) investigate the associations between primary exposure variables (disease category and funding) and the proportion of female enrollees, and (5) examine the associations of other clinical trial features with the proportion of female enrollees. Descriptive statistics were reported using medians and interquartile ranges (IQRs).

We examined the associations with the proportion of female enrollees using multivariable fractional regression analysis with a logit link.^22^ Regression analyses only included clinical trials with associated MeSH terms; therefore, 2495 clinical trials (12.5%) without MeSH terms were excluded from those analyses. To examine the association of disease category with the proportion of female enrollees, we used 2 regression models; model 1 included disease category, and model 2 included clinical trial features (without disease category). All analyses were 2-sided with a significance threshold of α = .05. Data were analyzed using R software, version 3.5.0 (R Foundation for Statistical Computing).

### Missing Data in Multivariable Regression Analysis

Each clinical trial feature had 0% to 7.9% missing data, which we addressed using multiple imputation by chained equations with 30 imputed data, with the assumption that missingness was random.^23^ Our analysis included pooled bayesian logistic regression models for binary data and pooled bayesian multinomial logistic regression models for categorical data (our sample did not have missing continuous data).

## Results

### Clinical Trial Population and Sex Reporting

**Figure 1.**
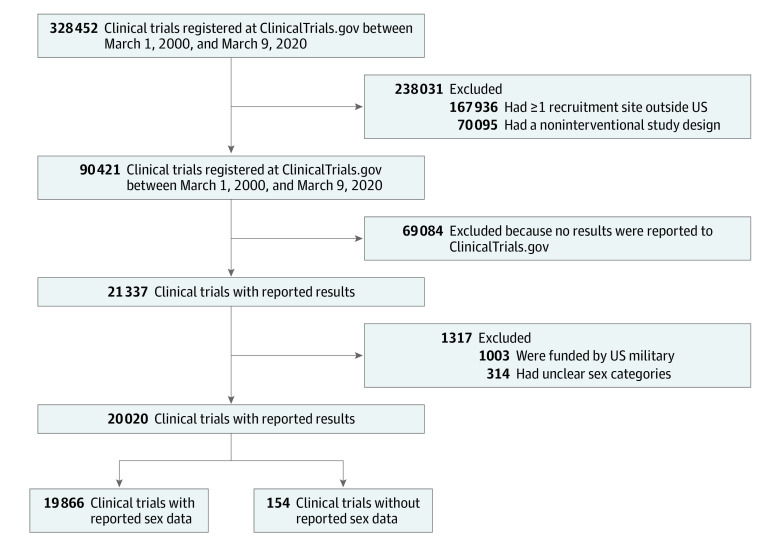
Flow Diagram of Clinical Trials Included in the Analysis

Of the 328 452 clinical trials registered between March 1, 2000, and March 9, 2020, in the ClinicalTrials.gov database, 21 337 clinical trials enrolling more than 5.11 million participants reported results. After excluding those with military funding or unclear sex categories, 20 020 clinical trials met inclusion criteria (Figure 1). Of those, 19 866 clinical trials (99.2%) reported sex.

### Sex Representation and Burden of Disease

**Figure 2.**
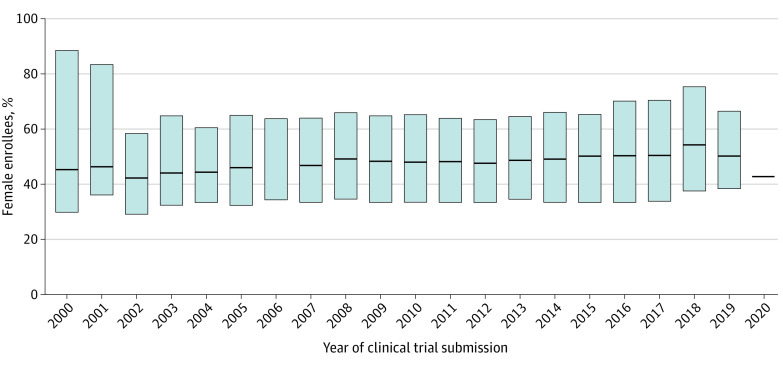
Median Enrollment of Female Participants in US Clinical Trials by Year Global proportion of female enrollment over time. Black lines represent the median proportion of female enrollees. Gray bars show interquartile ranges (IQRs). The IQR for 2020 could not be calculated because of the small number of clinical trials submitted to the ClinicalTrials.gov registry before March 2020.

Female representation across all clinical trials was approximately 50% between 2000 and 2020, with the lowest female representation occurring in 2002 (median, 41.1%; IQR, 29.2%-56.2%) and the highest occurring in 2018 (median, 60.0%; IQR, 40.6%-77.8%) (Figure 2).

**Figure 3.**
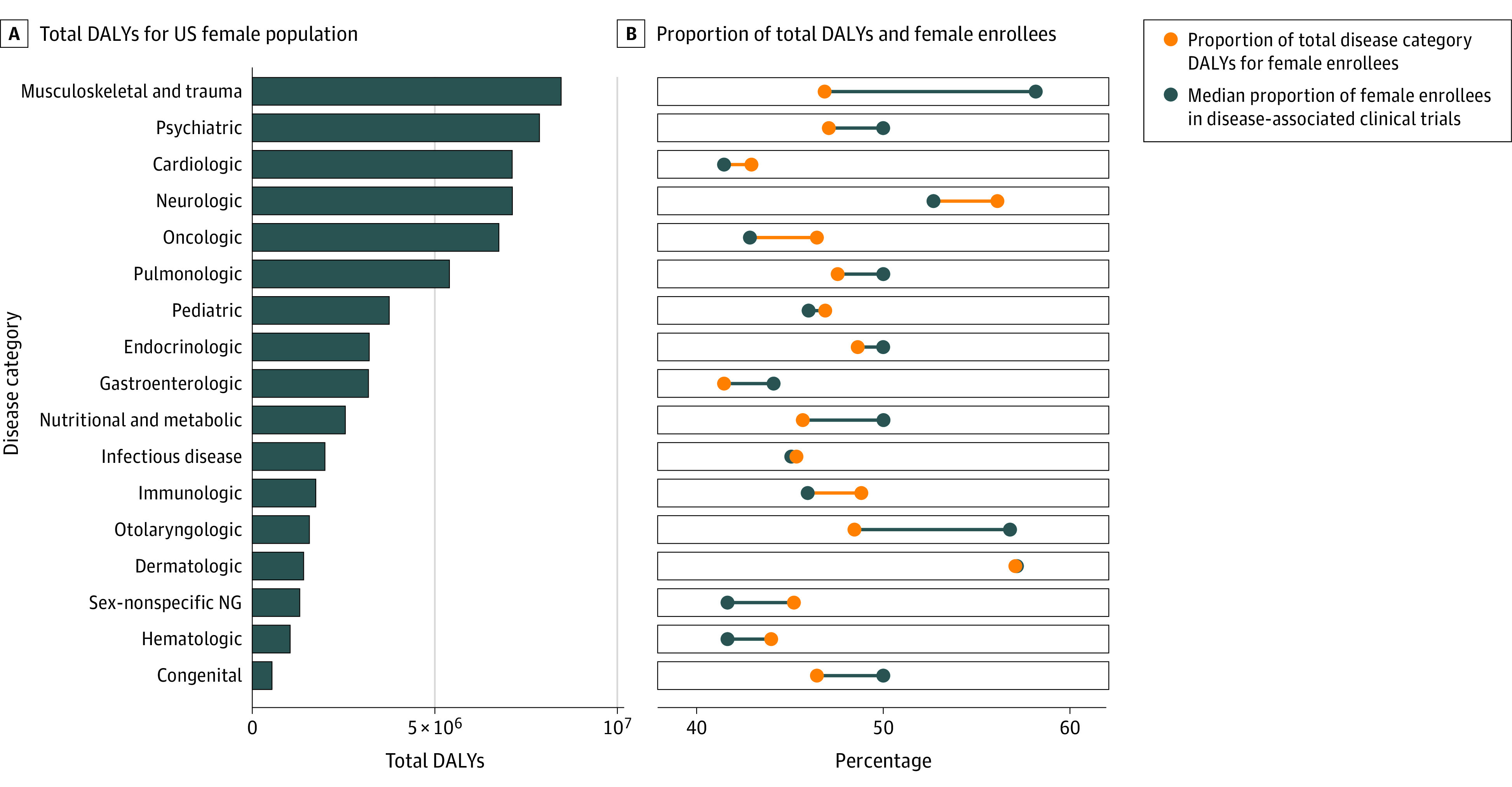
Burden of Disease, Female Proportion of Disability-Adjusted Life-Years (DALYs), and Female Proportion of Clinical Trial Enrollees by Disease Focus Lines connecting yellow and blue dots show the difference between the proportion of female participants and female DALYs. Blue lines indicate that the proportion of female participants is greater than the proportion of female DALYs. Yellow lines indicate that the proportion of female participants is less than the proportion of female DALYs. NG indicates nephrological and genitourinary.

Sex representation varied by disease category (Figure 3). Female participants comprised the smallest proportions of enrollees in cardiology (41.4%), sex-nonspecific nephrology and genitourinary (41.7%), and hematology (41.7%) clinical trials. Clinical trials in oncology had the greatest female underrepresentation relative to the female proportion of corresponding DALYs (42.9% vs 46.5%, respectively), followed by clinical trials in neurology (52.7% vs 56.1%), immunology (45.9% vs 48.8%), and sex-nonspecific nephrology and genitourinary disease (41.7% vs 45.2%).

The greatest disparities existed in disease categories in which male participants were underrepresented compared with their burden of disease (Figure 3; eTable 1 in the Supplement). Male participants were underrepresented in 8 disease categories, with the greatest disparity in clinical trials of musculoskeletal disease and trauma (11.3% difference between representation and proportion of DALYs). Only 46.9% of musculoskeletal disease and trauma DALYs were attributable to the female population, but 58.2% (IQR 40.0%-77.7%) of clinical trial participants were female. In otorhinolaryngology clinical trials, female enrollees comprised 56.8% (IQR, 42.2%-66.7%) of participants despite representing only 48.5% of DALYs. Although female enrollees represented 50% of clinical trial participants overall, they were overrepresented relative to their burden of disease in congenital (difference, −3.6%), nutritional and metabolic (difference, −4.3%), gastroenterology (difference, −2.7%), and psychiatry (difference, −2.9%) clinical trials. Dermatology and infectious disease clinical trials had relative parity (difference, −0.1% and 0.3%, respectively). Other disease foci indicated minor discrepancies (≤2% difference between proportion of DALYs and of female representation in clinical trials).

### Sex Representation and Clinical Trial Features

Several clinical trial features differed in female representation (Table 1). Although industry- and academic-funded clinical trials enrolled approximately equal proportions of male and female participants, US government–funded clinical trials had a median female enrollment of 46.7% (IQR, 30.8%-65.0%). Behavioral clinical trials enrolled a greater proportion of female participants (median, 56.7%; IQR, 40.7%-76.0%) than medical device clinical trials (median, 51.6%; IQR, 36.4%-70.6%). Female enrollees represented fewer than one-half of participants in clinical trials of biologic drugs and supplements (48.0%; IQR, 32.3%-65.6%), procedures (46.1%; IQR, 31.7%-64.6%), and other interventions (48.0%; IQR, 32.1%-66.7%). Clinical trial phase had the greatest variation in female enrollment; in phase 2/3 to phase 3 clinical trials, 51.7% (IQR, 38.9%-67.9%) of enrollees were female, and in phase 4 clinical trials, 51.1% (IQR, 36.4%-70.0%) were female. Phase 1 clinical trials had a median female enrollment of 42.9% (IQR, 27.8%-57.1%), and phase 1/2 to phase 2 clinical trials had a median female enrollment of 44.8% (IQR, 29.4%-62.5%).

**Table 1.** Proportion of Female Participants in Clinical Trials by Clinical Trial Feature

Feature	No.		Female proportion of participants, median (IQR), %
	Total studies	Total female participants	
Funding^a^			
Industry	9249	995 262	49.6 (33.3-65.6)
Academic	6647	470 397	50.0 (34.8-72.0)
US government	4124	886 371	46.7 (30.8-65.0)
Primary purpose			
Treatment	14 844	897 859	48.4 (33.0-66.7)
Basic scientific research	782	17 496	48.1 (29.3-64.8)
Other^b^	2364	834 096	51.7 (38.5-70.0)
Prevention	1514	476 769	53.6 (41.7-76.2)
Missing	516	105 810	45.9 (30.1-64.4)
Intervention			
Behavioral	1346	285 894	56.7 (40.7-76.0)
Medical device	3170	455 370	51.6 (36.4-70.6)
Biologic drugs or supplements	15 262	1 266 289	48.0 (32.3-65.6)
Procedure	1321	360 902	46.1 (31.7-64.6)
Other	3158	523 205	48.0 (32.1-66.7)
Phase			
Not applicable^c^	5800	1 294 097	52.5 (37.5-73.4)
1	1401	25 958	42.9 (27.8-57.1)
1/2-2	7582	235 049	44.8 (29.4-62.5)
2/3-3	2440	579 472	51.7 (38.9-67.9)
4	2797	197 454	51.1 (36.4-70.0)
Blinding			
None	11 606	1 129 049	46.9 (32.0-64.9)
Double	5944	601 746	50.7 (35.2-69.0)
Single	2452	541 042	54.2 (40.0-72.3)
Missing	18	60 193	50.5 (40.1-63.8)
Randomization			
Nonrandomized	8062	462 842	46.2 (30.8-64.7)
Randomized	11 854	1 865 245	50.0 (35.6-68.9)
Missing	104	3943	50.0 (33.3-65.2)
Overseen by a data safety monitoring committee			
No	10 135	1 221 154	50.0 (34.8-68.4)
Yes	8265	688 597	46.9 (31.6-66.0)
Missing	1620	422 279	50.0 (35.8-67.2)

Abbreviation: IQR, interquartile range.

^a^ Funding categories were determined through data on the sponsor and collaborators. Industry funding includes clinical trials with an industry sponsor or collaborating agency. US government funding includes remaining clinical trials with a US government sponsor or collaborating agency.

^b^ Other primary purposes include diagnostic, screening, supportive care, health services research, and other interventions.

^c^ In the ClinicalTrials.gov registry, not applicable is used to describe clinical trials without US Food and Drug Administration–defined phases, including clinical trials of medical devices or behavioral interventions.

### Clinical Trial Features Associated With Sex Representation

In model 1, which accounted for disease category, several disease categories were associated with lower female enrollment in the multivariable regression analysis (Table 2). Pediatric clinical trials had the most significant association with lower female enrollment (adjusted relative difference, −20.47%; 95% CI, −25.77% to −15.16%), followed by cardiology (adjusted relative difference, −18.68%; 95% CI, −22.87% to −14.47%) and infectious disease (adjusted relative difference, −18.51%; 95% CI, −24.25% to −12.76%) clinical trials. Less substantial associations were observed in clinical trials associated with gastroenterology (adjusted relative difference, −12.81%; 95% CI, −17.84% to −7.83%), sex-nonspecific nephrology and genitourinary disease (adjusted relative difference, −12.72%; 95% CI, −19.17% to −6.34%), psychiatry (adjusted relative difference, −11.29%; 95% CI, −15.80% to −6.81%), hematology (adjusted relative difference, −11.89%; 95% CI, −17.94% to −5.91%), and endocrinology (adjusted relative difference, −7.93%; 95% CI, −14.04% to −1.92%), which had negative associations with the proportion of female participants compared with all other clinical trials. No disease foci had a positive association with the proportion of female participants.

**Table 2.** Factors Associated With the Proportion of Female Participants Enrolled in Clinical Trials^a^

Factor	Relative difference (95% CI), %
Funding^b^	
Industry	1 [Reference]
Academic	2.38 (−0.73 to 5.44)
US government	0.14 (−3.45 to 3.66)
Primary purpose	
Treatment	1 [Reference]
Basic scientific research	0.58 (−6.89 to 7.75)
Other^c^	4.29 (0.11 to 8.35)
Prevention	8.48 (3.77 to 13.00)
Intervention	
All other clinical trials	1 [Reference]
Behavioral	4.17 (−1.84 to 9.94)
Medical device	−5.31 (−10.82 to 0.09)
Biologic drugs or supplements	−3.97 (−9.46 to 1.38)
Procedure	−1.54 (−6.61 to 3.40)
Other	−1.26 (−4.90 to 2.32)
Phase	
2/3-3	1 [Reference]
Not applicable^d^	1.44 (−3.56 to 6.29)
1	−5.43 (−12.18 to 1.15)
1/2-2	−3.07 (−7.48 to 1.26)
4	2.44 (−2.40 to 7.13)
Blinding	
None	1 [Reference]
Double	2.12 (−1.79 to 5.94)
Single	2.86 (−1.76 to 7.34)
Randomization	
Nonrandomized	1 [Reference]
Randomized	−1.33 (−6.76 to 3.96)
Oversight by data safety monitoring committee	
No	1 [Reference]
Yes	−1.30 (−3.92 to 1.28)
Disease focus^e^	
Musculoskeletal and trauma	2.76 (−2.82 to 8.14)
Psychiatry	−11.29 (−15.80 to −6.81)
Cardiology	−18.68 (−22.87 to −14.47)
Neurology	0.20 (−3.49 to 3.81)
Oncology	−1.29 (−5.40 to 2.74)
Pulmonology	−4.41 (−9.01 to 0.11)
Pediatrics	−20.47 (−25.77 to −15.16)
Endocrinology	−7.93 (−14.04 to −1.92)
Gastroenterology	−12.81 (−17.84 to −7.83)
Nutrition and metabolic	−2.14 (−7.70 to 3.28)
Infectious disease	−18.51 (−24.25 to −12.76)
Immunology	0.92 (−3.70 to 5.41)
Otorhinolaryngology	−2.90 (−10.46 to 4.40)
Dermatology	4.79 (−0.79 to 10.14)
Sex-nonspecific nephrology and genitourinary	−12.72 (−19.17 to −6.34)
Hematology	−11.89 (−17.94 to −5.91)
Congenital	−0.75 (−7.77 to 6.01)

^a^ Model 1 includes disease category.

^b^ Funding categories were determined through data on the sponsor and collaborators. Industry funding includes clinical trials with an industry sponsor or collaborating agency. US government funding includes remaining clinical trials with a US government sponsor or collaborating agency.

^c^ Other primary purposes include diagnostic, screening, supportive care, health services research, and other interventions.

^d^ In the ClinicalTrials.gov registry, not applicable is used to describe clinical trials without US Food and Drug Administration–defined phases, including clinical trials of medical devices or behavioral interventions.

^e^ Clinical trials could have more than 1 disease category. For analysis, each disease category was treated as a binary variable.

In the same model, the primary purpose of the clinical trial had the most significant association with the proportion of female enrollees. Clinical trials of preventive interventions and other primary purposes (including diagnostic, screening, supportive care, health services research, and other interventions) had adjusted relative differences of 8.48% (95% CI, 3.77%-13.00%) and 4.29% (95% CI, 0.11%-8.35%) in female enrollment, respectively, compared with clinical trials of treatment (Table 2). These associations were more substantial in model 1 relative to model 2, which did not account for disease category (eTable 2 in the Supplement). In contrast, although blinding and oversight by a data monitoring committee had significant associations with the proportion of female enrollees in model 2, these associations were not observed when disease category was included in model 1. Funding indicated no significant association with the proportion of female enrollees in either model.

## Discussion

This cross-sectional study of 20 020 US-based clinical trials found that, although male and female individuals had similar representation in all clinical trials conducted over the past 20 years, sex bias in clinical trial enrollment persists within medical specialties. Few previous studies have compared sex representation in clinical trials with burden of disease or have analyzed the association of clinical trial features with female representation across medical specialties. Compared with their respective burdens of disease, female participants are most underrepresented in oncology clinical trials, and male participants are most underrepresented in clinical trials of musculoskeletal disorders and trauma. Even after accounting for other clinical trial factors, disease category had the most significant association with lower female enrollment compared with other clinical trial features, particularly in the fields of cardiology, pediatrics, and infectious disease.

The present analysis allowed for the novel assessment of associations between different disease areas and female enrollment. The analyses found that female participants were underrepresented in oncology, neurology, immunology, urology, cardiology, and hematology relative to their disease burden. The finding that lower proportions of female participants were enrolled in clinical trials in cardiology and oncology is consistent with historical patterns in both fields.^7,24,25,26,27^ This finding is concerning because cardiologic and oncologic diseases are the leading causes of death among female individuals in the US.^25,26^ Furthermore, clinical trials provide an important mechanism for patients to access the best innovative therapies, particularly in the field of oncology. Underrepresentation of female participants in these clinical trials widens the previously documented sex gap in outcomes such as mortality.^25^ Parity in clinical trial enrollment can be considered a minimum standard, and efforts are needed to actively focus research on the female population. The historical underrepresentation of female participants in clinical trials is likely associated with the relative deficiency in knowledge of preventive strategies, disease manifestations, prognosis, and treatment of cardiovascular and neoplastic disease in the female population.^4,8^ This paradigm is also evident for obstetric conditions. Most pregnant women are excluded from clinical trials of diseases that may have consequences for their health,^28^ and clinical trials specifically focused on obstetric conditions comprise fewer than 2% of all clinical trials.^18^ A greater allocation of resources for female-focused clinical trials may be important to improving care and discerning the heterogenous manifestations of disease within the female population.

The present study highlights lesser-known disparities in immunology, sex-nonspecific nephrology and genitourinary disease, and hematology clinical trials. Despite the enrollment disparity observed in the descriptive analysis, these inequities were not associated with lower female enrollment in the multivariable analyses, suggesting that perhaps lower female enrollment is associated with other clinical trial factors. These disease areas warrant further investigation to identify the reasons that female enrollees represent a minority of clinical trial participants. Sex differences across these fields are well documented and important to developing beneficial preventive, diagnostic, and treatment strategies. Increased parity, in combination with sex-specific analysis, could elucidate these differences and provide the best approach to reducing corresponding DALYs for each sex.

This study also presents novel findings on male underrepresentation in clinical trials. Male enrollees comprised a minority of participants in 3 disease categories and were underrepresented compared with their disease burden in 8 disease categories. This imbalance is consistent with the observations of a previous study, which indicated that sex bias may have negative implications for both sexes.^29^ The finding that clinical trials of preventive interventions were independently associated with greater female enrollment is consistent with the established paradigm regarding sex-specific use of preventive services and access to health care.^29^ Identifying areas of research in which sex bias disadvantages male individuals is important to improving population health. The present study’s finding of male underrepresentation in mental health and trauma research assumes greater importance in American society, in which suicide, violence, and substance use are increasingly associated with higher morbidity and premature mortality in the male population.^30^

This study included an innovative multivariable analysis of the clinical trial features associated with female enrollment across medical specialties. Previous univariable, often single-specialty, analyses suggested that there were associations between funding, randomization, study phase, number of sites, intervention type, and enrollment.^10,13,25^ One previous study compared sex bias with the prevalence of disease among global studies registered in the ClinicalTrials.gov database; however, the study used a limited analysis that controlled only for registry submission year and disease category.^24^ The novel findings of the present study may reflect the larger sample, longer study period, or different dynamics within medical fields. For example, within cardiology clinical trials, several studies indicated that female enrollment varied with individual diseases and funding for those diseases.^10,13,25,31^ Studies from other fields have similarly reported that clinical trial features may vary within disease foci.^31,32^ Medical fields with more balanced sex representation may serve as a model for decreasing sex bias in clinical trials.

The consistently high rates of sex reporting in clinical trials are encouraging. The current study found higher rates of sex reporting compared with a previous study of clinical trial publications from the same period.^31^ Discrepancies between reporting in the ClinicalTrials.gov registry and published results represent an important gap; given that registry reporting indicates that sex data exist, these data can be included in clinical trial publications and used for sex-specific analysis. Some medical journals do not require sex reporting or sex-specific analysis for publication.^33^ We propose that sex reporting represents an important step toward equitable enrollment and examination of sex-specific pathophysiologic disease factors and treatment.

Despite the high rates of sex reporting in the ClinicalTrials.gov registry, a meaningful analysis of the representation of gender was not possible because of the small number of clinical trials that included and reported on nonbinary genders or transgender health, highlighting a need for greater inclusion of gender diversity in medical research. A standardized system that includes all sexes and genders, including transgender and nonbinary genders, in reporting is necessary to improve health for all. The relative absence of the gender nonbinary and transgender community from clinical trials limits medical progress for these communities.

### Strengths and Limitations

This study has several strengths. A 2018 study has called for the comparison of disease burden with sex representation as the next step in understanding sex bias in clinical research.^13^ Our study assesses 2 databases that accurately capture the entire US landscape of clinical trials and burden of disease, avoiding the sampling biases found in previous studies by comparing US data from the ClinicalTrials.gov registry with global disease burden. Although previous investigations narrowed their focus to specific diseases,^7,31,32,34,35^ the present study provides novel data, assessing and comparing 17 disease categories. This study’s distinct multivariable analysis examines potential factors, including disease category, that may be associated with sex bias among all US-based clinical trials. The study also elucidates important factors that may be negatively associated with male health, an underexamined area of sex bias.

This study also has limitations. The study does not address whether potential participants (male or female) were approached for study participation. In addition, the point in the clinical trial process at which barriers to enrollment occur is not clear. Database limitations within the ClinicalTrials.gov registry also exist. Most clinical trials do not report results, presenting a possible selection bias and preventing extrapolation to the entirety of clinical research. The National Library of Medicine does not require or enforce valid data entry in the ClinicalTrials.gov registry, and quality reviews may only identify a small proportion of data errors.^36^ Other established limitations include changes to the database over time and heterogeneity in data entry. In addition, disease prevalence may have changed over the study period, and the GBD data account for only a portion of the years examined.

## Conclusions

Professional and governmental organizations have mobilized numerous efforts to increase female representation in clinical trials over the past 30 years.^9^ Although sex equity has progressed, sex bias persists within medical fields, with potentially negative consequences for all individuals. Sex reporting and sex-specific analysis are warranted for all research bodies. Furthermore, efforts are needed to ensure that sex representation better reflects the health burdens of the population. Without sex-specific analysis and increased parity, the interpretation of data for both male and female populations remains unreliable,^8^ and the benefits of research may not reach both groups.^27,37^ Analysis of the most important factors associated with sex bias within medical fields may reveal areas for improvement and mechanisms to increase inclusivity so that the research represents and serves both sexes.

## Notes

Supplement. **eMethods 1.** Clinical Trial Disease Category and Medical Subject Heading (MeSH) Categories **eMethods 2.** Global Burden of Disease Data and Medical Subject Heading (MeSH) Categories **eTable 1.** Comparison of Female Burden of Disease and Female Enrollment by Disease Category **eTable 2.** Factors Associated With the Proportion of Female Participants Enrolled in Clinical Trials
