# Supplementary material for: Analysis of Female Enrollment and Participant Sex by Burden of Disease in US Clinical Trials Between 2000 and 2020
Source: JAMA Netw Open. 2021 Jun 18;4(6):e2113749. doi: 10.1001/jamanetworkopen.2021.13749 (PMC8214160; doi:10.1001/jamanetworkopen.2021.13749)
Supplement: Supplement. — eMethods 1. Clinical Trial Disease Category and Medical Subject Heading (MeSH) Categories eMethods 2. Global Burden of Disease Data and Medical Subject Heading (MeSH) Categories eTable 1. Comparison of Female Burden of Disease and Female Enrollment by Disease Category eTable 2. Factors Associated With the Proportion of Female Participants Enrolled in Clinical Trials [file jamanetwopen-e2113749-s001.pdf]

Supplementary Online Content

Steinberg JR, Turner BE, Weeks BT, et al. Analysis of female enrollment and participant sex by burden of disease in US clinical trials between 2000 and 2020. *JAMA Netw Open*. 2021;4(6):e2113749. doi:10.1001/jamanetworkopen.2021.13749

eMethods 1. Clinical Trial Disease Category and Medical Subject Heading (MeSH) Categories

eMethods 2. Global Burden of Disease Data and Medical Subject Heading (MeSH) Categories

eTable 1. Comparison of Female Burden of Disease and Female Enrollment by Disease Category

eTable 2. Factors Associated With the Proportion of Female Participants Enrolled in Clinical Trials

This supplementary material has been provided by the authors to give readers additional information about their work.

© 2021 Steinberg JR et al. *JAMA Network Open*.

eMethods 1. Clinical Trial Disease Category and Medical Subject Heading (MeSH) Categories

Trial disease category was determined using the MeSH terms assigned in the trial record. Disease category is non-exclusive for two reasons:

1. MeSH tree structure permits the same term to be accessed using multiple branches. For example, the MeSH term "Stroke" can be accessed through the Cardiology branch using [C14.907.253.855] or through the Neurology branch using [C10.228.140.300.775].[36]
2. The disease category for a trial assigned the MeSH term "Stroke" would thus be labeled both Cardiology and Neurology.
3. The National Library of Medicine (NLM) may apply multiple MeSH terms to each trial record as relevant. For example, the trial NCT00000124 investigating therapies for patients with ocular melanoma was assigned the MeSH terms "Choroid neoplasms" and "Uveitis", which correspond to Oncology and Eye Disease categories.

In most cases our disease categories are identical to their respective MeSH branching's. To improve clarity, we made the following modifications to the MeSH branching:

- **Female Urogenital; Male Urogenital; Genitourinary:** The MeSH branches "Male Urogenital Diseases" and "Female Urogenital Diseases and Pregnancy Complications" have significant overlap, including some diseases which are sex-neutral. For example "Nephritis" can be accessed using both [C12.777.419.570] and [C13.351.968.419.570]. However, some terms are specific to the female sex (e.g. "Adnexal Diseases", [C13.351.500.056]) and others are specific to the male sex (e.g.

© 2021 Steinberg JR et al. *JAMA Network Open*.

"Balanitis", [C12.294.494.136]). We manually reviewed all MeSH terms within either the "Male Urogenital Diseases" or the "Female Urogenital Diseases and Pregnancy Complications" branch and reassigned them into one of three categories: (1) "Female Urogenital" for diseases specific to the female sex; (2) "Male Urogenital" for diseases specific to the male sex; (3) "Genitourinary" for diseases that are not specific to either sex.

- **Musculoskeletal and Trauma:** All diseases in the MeSH branch "Wounds and Injury" were manually reviewed. Diseases consistent with musculoskeletal processes (e.g. "Ankle Fractures", [C26.404.014]) or trauma (e.g. "Post-Concussion Syndrome", [C26.915.300.450.500.500]) were combined with diseases in the "Musculoskeletal Diseases" branch to create the category "Musculoskeletal and Trauma".
- **Otorhinolaryngology:** The MeSH branches "Stomatognathic Diseases" and "Otorhinolaryngologic Diseases" were combined to create the category "Otorhinolaryngology".
- **Congenital:** The MeSH branch "Congenital, Hereditary, and Neonatal Diseases and Abnormalities" was abbreviated to "Congenital" but was otherwise unchanged.
- **Pediatric:** Because diseases affecting pediatric populations are frequently studied in trials run by pediatric subspecialists, we separated these trials as their own disease focus. There is no MeSH term used that explicitly labels trials of pediatric populations. While some diseases (e.g. croup) are mostly associated with pediatrics, these diseases can sometimes be encountered in adult populations and may be an important topic of study for a clinical trial. Many other diseases can be commonly seen in both pediatric and adult populations (e.g. epilepsy, leukemia, urinary tract infections). We instead identified our

© 2021 Steinberg JR et al. *JAMA Network Open*.

pediatric trials using participant eligibility criteria. Any trial with a maximum eligibility of 18 or fewer years of age were assigned the category “Pediatric”. We subsequently removed any other disease category previously associated with a Pediatric trial to ensure that all other MeSH categories reflect only adult (i.e. individuals aged 19 years or older) trials.

- **Other Disease:** Any MeSH term which was not otherwise associated with a major MeSH disease category was assigned to the category “Other Disease”.

© 2021 Steinberg JR et al. *JAMA Network Open*.

eMethods 2. Global Burden of Disease Data and Medical Subject Heading (MeSH) Categories

We accessed disease burden data from the global burden of disease (GBD) project using the Global Health Data Exchange (http://ghdx.healthdata.org/gbd-results-tool).[37] Disease "cause" is defined by GBD as "A single disease or injury or an aggregation of diseases and injuries that causes death or disability." They define Disability-Adjusted Life Years (DALYs) as "The sum of years lost due to premature death (YLLs) and years lived with disability (YLDs). DALYs are also defined as years of healthy life lost." We downloaded all data on DALYs for the year 2017 for all 360 disease causes measured by the GBD. We manually reviewed each disease cause and mapped it to any of the appropriate MeSH categories (including modifications previously described).

Though GBD causes are intended to be mutually exclusive, MeSH terms are overlapping within the MeSH tree. Thus, where appropriate, GBD causes were mapped to multiple MeSH categories. For example, within GBD the cause “Rheumatic heart disease” is hierarchically under the cause “Cardiovascular diseases” and not the cause “Communicable, maternal, neonatal, and nutritional diseases,” however within MeSH we mapped "Rheumatic heart disease" to both the “Infections” category and the “Cardiovascular Diseases” category. Of note, the comparable MeSH term “Rheumatic Heart Disease” is located both within the “Infections” branch ([C01.150.252.410.890.731.649]) and the “Cardiovascular Diseases” branch ([C14.280.874]). Not all GBD causes have exact correlates in MeSH. In such cases we used our best judgment in mapping to the appropriate categories.

© 2021 Steinberg JR et al. *JAMA Network Open*.

GBD does not contain a specific cause for pediatric diseases. We stratified all cause DALYs using the “Age group” field to determine the proportion of DALYs for each cause that were attributable to persons under the age of 20. The total such DALYs across all causes were summed to calculate the total DALYs attributable to pediatric disease burden. These DALYs were also subtracted from each of their respective causes so that the remaining DALYs for each cause were only those associated with adult disease burden (i.e. individuals aged 20 and older). Only the adult DALYs were used to calculate disease burden for each of the various MeSH categories. All of the pediatric DALYs were assigned to the previously described MeSH category that we created for pediatrics.

© 2021 Steinberg JR et al. *JAMA Network Open*.

eTable 1. Comparison of Female Burden of Disease[a] and Female Enrollment by Disease Category

Disease Category	DALYs in the Female Population	Female Percent of DALYs	Median Proportion of Female participants	Difference between female DALY percent and female participation percent
Cardiology	7128359.466	42.9%	41.5%	1.5%
Congenital	549052.7848	46.4%	50.0%	-3.6%
Dermatology	1401854.481	57.1%	57.1%	-0.1%
Endocrinology	3201910.79	48.6%	50.0%	-1.4%
Otorhinolaryngology	1564030.916	48.5%	56.8%	-8.3%
Digestive System	3188170.457	41.5%	44.1%	-2.7%
Hematology	1035748.682	44.0%	41.7%	2.3%
Immunology	1746411.78	48.8%	45.9%	2.9%
Infectious disease	1978436.118	45.3%	45.1%	0.3%
Psychiatry	7860668.269	47.1%	50.0%	-2.9%
Musculoskeletal and Trauma	8459052.278	46.9%	58.2%	-11.3%
Neurology	7125829.599	56.1%	52.7%	3.4%
Nutritional and Metabolic	2543911.62	45.7%	50.0%	-4.3%
Oncology	6758535.765	46.5%	42.9%	3.6%
Other			53.7%	
Pediatric	3751215.325	46.9%	46.0%	0.9%
Pulmonology	5395649.045	47.6%	50.0%	-2.4%
Sex nonspecific nephrology and genitourinary	1296542.613	45.2%	41.7%	3.5%

a. Measured in DALYs: Disability Adjusted Life Years

© 2021 Steinberg JR et al. *JAMA Network Open*.

eTable 2. Factors Associated With the Proportion of Female Participants Enrolled in Clinical Trials[a]

Variable		Relative Percent Difference	(95% Confidence Interval)		P Value
Primary Purpose					
	Treatment	Reference			
	Basic Science	-0.51%	(-8.95%,	7.80%)	0.9
	Other[b]	3.16%	(-1.59%,	7.85%)	0.1
	Prevention	7.23%	(1.88%,	12.47%)	0.008
Intervention					
	Behavioral	3.47%	(-3.34%,	10.15%)	0.3
	Device	-4.42%	(-10.49%,	1.62%)	0.2
	Drug Biologics or Supplements	-3.79%	(-9.83%,	2.20%)	0.2
	Procedure	-1.32%	(-6.89%,	4.20%)	0.6
	Other	-1.97%	(-5.96%,	2.00%)	0.3
Phase					
	Phase 2/3-3	Reference			
	Not applicable [c]	3.39%	(-2.23%,	8.93%)	0.2
	Phase 1	-6.62%	(-14.05%,	0.78%)	0.07
	Phase 1/2-2	-4.53%	(-9.34%,	0.26%)	0.06
	Phase 4	2.36%	(-3.13%,	7.78%)	0.4
Number of arms					
	1	Reference			
	≥2	-0.71%	(-6.25%,	4.78%)	0.8
Estimated Enrollment					
	100-499	Reference			
	0-9	-7.74%	(-13.24%,	-2.26%)	0.005
	0-49	-5.38%	(-9.49%,	-1.29%)	0.01
	50-99	-3.21%	(-7.74%,	1.29%)	0.1
	500-999	3.62%	(-4.97%,	12.00%)	0.4
	≥1000	-0.28%	(-10.44%,	9.68%)	0.9
Year					
	The previous year	Reference			
	Each Additional Year	-0.05%	(-0.47%,	0.37%)	0.8
Funding[d]					
	Industry	Reference			
	Academic	2.82%	(-0.69%,	6.29%)	0.1
	US Government	-1.30%	(-5.28%,	2.64%)	0.5
Blinding					

© 2021 Steinberg JR et al. *JAMA Network Open*.

	None	Reference			
	Double	5.08%	(0.85%,	9.26%)	0.02
	Single	5.57%	(0.39%,	10.66%)	0.04
Randomization					
	Nonrandomized	Reference			
	Randomized	-1.57%	(-7.56%,	4.36%)	0.6
Oversight by a Data Safety Monitoring Committee					
	No	Reference			
	Yes	-3.13%	(-6.03%,	-0.24%)	0.03
Number of Facilities					
	1	Reference			
	≥2	0.75%	(-2.39%,	3.86%)	0.6

a. Model 2 excluding disease focus.
b. Other primary purposes include diagnostic, screening, supportive care, health services research and other.
c. On ClinicalTrials.gov "Not Applicable" is used to describe trials without Food and Drug Administration-defined phases, including trials of devices or behavioral interventions
d. Funding categories were determined with data on the sponsor and collaborators. Industry funding includes trials with an industry sponsor or collaborating agency; US Government trials include remaining trials with a US Government sponsor or collaborating agency.

© 2021 Steinberg JR et al. *JAMA Network Open*.
